# Early-Onset Neonatal Meningitis Caused by an Unusual Pathogen‐*Moraxella catarrhalis*

**DOI:** 10.1155/2019/4740504

**Published:** 2019-01-09

**Authors:** Shraddha Siwakoti, Sohani Bajracharya, Neetu Adhikaree, Rinku Sah, Rupa Singh Rajbhandari, Basudha Khanal

**Affiliations:** ^1^Assistant Professor, Department of Microbiology, BP Koirala Institute of Health Sciences, Dharan, Nepal; ^2^Junior Resident, Department of Microbiology, BP Koirala Institute of Health Sciences, Dharan, Nepal; ^3^Professor, Department of Pediatrics, BP Koirala Institute of Health Sciences, Dharan, Nepal; ^4^Professor and Head, Department of Microbiology, BP Koirala Institute of Health Sciences, Dharan, Nepal

## Abstract

**Introduction:**

*Moraxella catarrhalis* are part of the normal flora of the human respiratory tract and are known to have low pathogenic potential. The organism is rarely reported in the literature as the causative agent of meningitis. We report the first case of early-onset neonatal meningitis associated with *M. catarrhalis* from Nepal.

**Case Report:**

A 3-day-old baby with fever and yellowish discolouration of the body since 48 hrs was admitted to the neonatal ward. The baby developed 3 episodes of seizures in the form of uprolling of eyes on the first day of admission during phototherapy course for raised serum bilirubin. Sepsis screen was positive, and meningitis was confirmed as the cerebrospinal fluid culture grew *M. catarrhalis.* Cranial ultrasound scan was normal. The baby received a 21-day course of intravenous cefotaxime and amikacin. Recovery has been uneventful to date.

**Conclusion:**

Neonatal meningitis is a life-threatening infection. This case report presents an uncommon aetiology of neonatal meningitis which can be misidentified in the diagnostic bacteriology laboratory in resource constraints area like ours.

## 1. Introduction

Neonatal meningitis causes substantial morbidity and mortality and is commonly caused by Group *B Streptococcus*, *Streptococcus pneumoniae*, and *Haemophilus influenzae* in Nepal [1]. *Moraxella (Branhamella) catarrhalis*, formerly called *Neisseria catarrhalis*, is an aerobic, Gram-negative coccobacillus frequently found as a commensal of the upper respiratory tract [2]. They were long considered to have low pathogenic potential [3] and are rarely reported causing invasive infections in humans. They are unusual organism to be isolated from cerebral spinal fluid. We report a case of early-onset neonatal meningitis with *M. catarrhalis*, most likely due to vertical transmission.

## 2. Case Report

**Figure 1:**
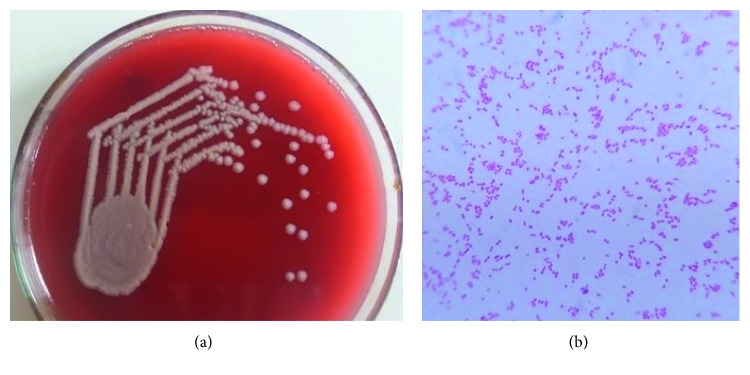
(a) Growth of *M. catarrhalis* on a blood agar. (b) Gram staining of the organism.

A 3-day-old baby was admitted to the neonatal ward due to fever and yellowish discolouration of the body since 48 hrs. On examination, the baby was irritable, jaundiced with heart rate 140/min, respiratory rate 42/min, and temperature 37.8°C. The child was born full term without complications by normal delivery to a 21-year-old primigravida woman with an unremarkable antenatal period. The neonate's weight, length, and cranial circumference at birth were 3155 g, 50 cm, and 34 cm, respectively. APGARs scores were 8 and 9, at 1 and 5 minutes of birth. On the current admission, the baby was irritable and jaundiced on physical examination and his heart rate was 140/min, respiratory rate was 42/min, and the temperature was 37.8°C. Sepsis screen was positive (C-reactive protein, 4.06 mg/dL; total leucocyte count, 15 700 cells/mm^3^ of blood and presence of toxic granules on peripheral blood smear). Total serum bilirubin was 15.2 *µ*g/dL. Phototherapy was started, and during its course on the first day of admission (DOA), the baby developed 3 episodes of seizures in the form of uprolling of eyes. The cerebrospinal fluid (CSF) analysis revealed an elevated white cell count of 300 × 10^6^ cells/L (95% polymorphs and 5% lymphocytes), raised protein level of 620 mg/L, and decreased glucose level of 1.9 mmol/L. Gram stain showed few pus cells. CSF culture on chocolate and blood agar grew white, opaque, and smooth 0.5 to 1 mm colonies (Figure 1), whereas in MacConkey's agar, non‐lactose fermenting bacillus was obtained after standard incubation. Gram staining of the isolate revealed Gram-negative coccobacilli (Figure 1). It produced catalase, oxidase, and DNase, reduced nitrate and nitrite, hydrolysed tributyrin, and did not ferment glucose, maltose, lactose, and sucrose. The growth was identified as *Moraxella catarrhalis*. Urine and blood cultures were negative. Cranial ultrasound scan was normal. All other laboratory parameters were unremarkable. The child was empirically treated with IV amikacin and cefotaxime, and it was continued for 21 days after obtaining the antibiotic sensitivity report. The baby was discharged from hospital in good clinical condition on 22nd DOA.

## 3. Discussion

*Moraxella catarrhalis* are non‐motile Gram-negative coccobacilli which are obligate aerobes, asaccharolytic, and oxidase producer. They are part of the normal flora of the human respiratory tract and are infrequently isolated from clinical specimens.

*M. catarrhalis* have been commonly implicated as etiologic agents of otitis media and sinusitis in children but it rarely causes bacteremic illness [3]. Isolated *M. catarrhalis* neonatal meningitis is an uncommon clinical finding. Literature review revealed a fifteen day-old boy with *M. catarrhalis* meningitis with good outcome after antibiotic treatment [4]. One case of fatal meningitis in an immunocompromised neonate due to *M. catarrhalis* has been reported [5]. Here, *M. catarrhalis* were isolated from the CSF of a neonate. The isolate was identified based on the cultural characteristics, Gram staining, and biochemical tests. The identity of *M. catarrhalis* is best confirmed by positive reactions for DNAase production, reduction of nitrate and nitrite, and tributyrin hydrolysis [6], and all these three tests were positive in our case. Despite the fact that a high percentage of *M. catarrhalis* strains producing *β*-lactamase have been reported since the 1970s, *M. catarrhalis* remains universally sensitive to most antibiotics [7]. Our isolate was a *β*-lactamase non‐producer, and the baby had a good clinical outcome without complications after IV cefotaxime and amikacin. A similar case of neonatal meningitis with *M. catarrhalis* reported by Fakih and Daakour also had the alike findings of complete resolution of symptoms without neurologic sequels after receiving the full course of IV ceftazidime [4]. In our case, the early onset of symptoms in a newborn allows us to hypothesize a vertical transmission of the bacteria. The present case has a limitation that the definitive identification of the species of the organism with genetic techniques could not be performed due to limited resources.

## 4. Conclusion

Neonatal meningitis is a life-threatening infection. We report a rare neonatal meningitis case due to an unusual agent *M. catarrhalis,* which can be misidentified in the diagnostic bacteriology laboratory in resource constraints area like ours.
